# Hints for Prospective Followers of the Newcastle Plan

**Published:** 1914-10-31

**Authors:** 


### HINTS FOR PROSPECTIVE FOLLOWERS OF THE NEWCASTLE PLAN.

Anyone who may wish to form an idea of the cost per bed of the Newcastle style of buildings should estimate somewhere as follows:—For two wards of twenty-six beds each, building, heating, lighting, &c., including sanitary and bath accommodation, between £1,300 and £1,400, and furnishing need not bring the cost to much more than £2,000. Of course, each hospital can furnish in any style it likes, but the house governor is satisfied that the wards could be built and reasonably furnished for this sum. The great point of these buildings is to keep them well painted. One which the infirmary has had for five years and which has been kept well painted shows no sign anywhere of the iron going. The fact that steam has been taken for heating off the infirmary's existing boilers makes very little difference in the initial cost, as the buildings could be satisfactorily heated by a separate small boiler and furnace. Newcastle avoided this because it would be slightly more troublesome to look after and more expensive in the long run.

